# SLC39A5 promotes the malignant progression of gastric cancer by activating BATF phosphorylation

**DOI:** 10.1016/j.jbc.2025.110754

**Published:** 2025-09-22

**Authors:** Xinyu Wang, Tao Li, Mingxi Xia, Leichao Sun, Xudong Wang

**Affiliations:** 1 Department of Gastrointestinal Nutrition & Hernia Surgery, The Second Hospital of Jilin University, Changchun, Jilin Province, P.R. China; 2 Department of Colorectal & Anal Surgery, The Second Hospital of Jilin University, Changchun, Jilin Province, P.R. China

**Keywords:** SLC39A5, gastric cancer, PIM1, HIP1R, BATF

## Abstract

Despite advances in diagnostic and therapeutic methods for gastric cancer (GC), early detection continues to be a significant challenge, resulting in late-stage diagnoses and poor survival outcomes. Studies have shown that solute carrier family 39 member 5 (SLC39A5) is upregulated in GC and may serve as a potential prognostic biomarker. However, the exact role of SLC39A5 and its underlying mechanisms remains unclear. To evaluate cell proliferation, migration, and invasion, a variety of assays, including Cell Counting Kit-8, 5-ethynyl-2′-deoxyuridine incorporation, scratch, and Transwell assays, were conducted. The molecular interactions among genes were investigated through coimmunoprecipitation, chromatin immunoprecipitation, and dual luciferase reporter assays. An *in vivo* GC mouse model was established to substantiate our *in vitro* findings. Knockdown of SLC39A5 inhibited GC cell proliferation, migration, and invasion. Furthermore, SLC39A5 increased Moloney murine leukemia virus 1 (proto-oncogene serine/threonine-protein kinase 1 [PIM1]) kinase activity by enhancing zinc influx, which in turn triggered basic leucine zipper ATF-like transcription factor (BATF) phosphorylation and stabilized BATF protein. BATF overexpression reversed the inhibitory effect of SLC39A5 depletion on the behavior of GC cells and tumor growth. Moreover, we found that BATF, combined with the Jun proto-oncogene, AP-1 transcription factor subunit (JUN), led to the suppression of Huntingtin interacting protein 1–related (HIP1R) expression and the activation of the PI3K/protein kinase B (AKT) pathway. In conclusion, SLC39A5 promotes the progression of GC *via* the BATF–HIP1R axis, which suggests that SLC39A5 acts as a therapeutic or diagnostic target for GC.

Gastric cancer (GC) is a common solid tumor with a high mortality rate, currently standing as the fifth most frequent cause of cancer-related deaths globally (1). Despite advancements in diagnostic techniques and therapeutic interventions, early symptoms of GC are subtle, and effective early screening methods are lacking, leading to most diagnoses occurring at advanced stages with poor survival outcomes (2). The development and progression of GC involve the dysregulation of multiple signaling pathways, cytokines, and epigenetic alterations (3). However, the precise mechanisms underlying GC remain incompletely understood. Therefore, there is an urgent need to enhance our understanding of GC pathogenesis to develop more accurate methods for early diagnostic techniques, prognostic assessments, and effective therapeutic approaches.

Solute carrier family 39 member 5 (SLC39A5), a protein belonging to the SLC39 family, plays a role in multiple physiological processes, including enzyme activation, cell growth, and apoptosis (4). Recent studies have highlighted the relevance of SLC39A5 in GC and other malignancies (5, 6). For example, Ding *et al.* (5)reported that SLC39A5 was elevated in GC and associated with the overall survival rate of GC patients, suggesting its potential as a prognostic biomarker. Similarly, Liu *et al.* (7)indicated that SLC39A5 enhanced the proliferation of lung adenocarcinoma cells by activating the PI3K/protein kinase B (AKT) signaling pathway. Despite these findings, the functional role and the underlying mechanism of SLC39A5 in GC remain unclear. SLC39A5 functions as a zinc (Zn) transporter, playing a key role in maintaining intracellular Zn homeostasis, which is essential for normal physiological functions (8). By facilitating the influx of extracellular Zn^2+^ into the cytoplasm, SLC39A5 contributes directly to this homeostatic balance (9). Importantly, dysregulation of Zn^2+^ transport can lead to Zn imbalance, a phenomenon increasingly associated with cancer progression (10). Thus, elucidating the role of SLC39A5 in Zn^2+^ transport in GC may provide critical insights into its potential contribution to cancer development and progression.

Zn transport activity is closely related to kinase signaling pathways. For example, both Zn excess and deficiency have been associated with extracellular signal–regulated kinase–dependent cell death in neuronal systems (11, 12). In addition, elevated Zn levels or Zn ionophores, such as Zn pyrithione, can activate other kinase pathways, including stress-related kinases, c-Jun N-terminal kinase and p38 mitogen–activated protein kinase, as well as Src family kinases, PKC and the Zn-sensing G protein–coupled receptor 39 (13, 14, 15, 16). These findings highlight a strong mechanistic link between Zn^2+^ transporter activity and kinase signaling. The proviral integration site for Moloney murine leukemia virus (proto-oncogene serine/threonine-protein kinase [PIM]) kinases constitutes a family of highly conserved serine/threonine kinases comprising three isoforms: PIM1, PIM2, and PIM3 (17). These kinases are involved in regulating a range of cellular processes, including proliferation, survival, and metabolism (18). The aberrant expression of PIM kinases has been reported across multiple human cancers, with distinct isoform-specific expression patterns identified in GC (18). Notably, PIM1 and PIM3 were frequently upregulated in GC tumor tissues and have been associated with poor prognosis and increased tumor aggressiveness (19, 20). Pharmacological inhibition of PIM kinases has been demonstrated to exert antitumor effects in GC models (21), indicating their potential as therapeutic targets. However, whether SLC39A5 regulates GC progression by transporting Zn^2+^ and then affecting PIM kinase activity remains unclear.

Basic leucine zipper ATF-like transcription factor (BATF) is a member of the AP-1 transcription factor family and is involved in regulating gene expression, particularly in immune cells (22). It plays a key role in T-cell differentiation and function by modulating cytokine signaling pathways (23). BATF was documented to be an oncogene in multiple types of cancer, including colorectal cancer, non–small cell lung cancer, and others (24, 25). Gene Expression Profiling Interactive Analysis (GEPIA) revealed that BATF expression was upregulated in GC. The aforementioned suggests that BATF may play a carcinogenic role in GC. BATF can form transcriptional repressive, DNA-binding heterodimers with Jun proto-oncogene, AP-1 transcription factor subunit (JUN) proteins (26). These BATF–JUN dimers play significant roles in regulating downstream gene expression, such as IRF4 transcription in T cells (26). Importantly, BATF undergoes phosphorylation at multiple serine, threonine, and at least one tyrosine residue (27). Interestingly, through bioinformatics prediction, we found that BATF only has the possibility of protein binding with PIM1 in the PIM family. In addition, inhibition of PIM1 has been reported to reduce the stability of its downstream effectors (28). The aforementioned suggests that BATF protein stability may be regulated by PIM1-mediated phosphorylation.

Thus, this study aims to elucidate the molecular mechanism by which SLC39A5 influences GC progression through Zn homeostasis and kinase activity. Specifically, we hypothesized that SLC39A5 enhanced PIM kinase activity by enhancing Zn influx, leading to BATF phosphorylation and stabilization. This promoted BATF–JUN heterodimer formation and its transcriptional inhibitory effect on downstream genes, ultimately advancing GC progression. This study may provide new insights into the molecular underpinnings of GC and potentially unveil new therapeutic targets for intervention.

## Results

### SLC39A5 was upregulated in GC

**Figure 1:**
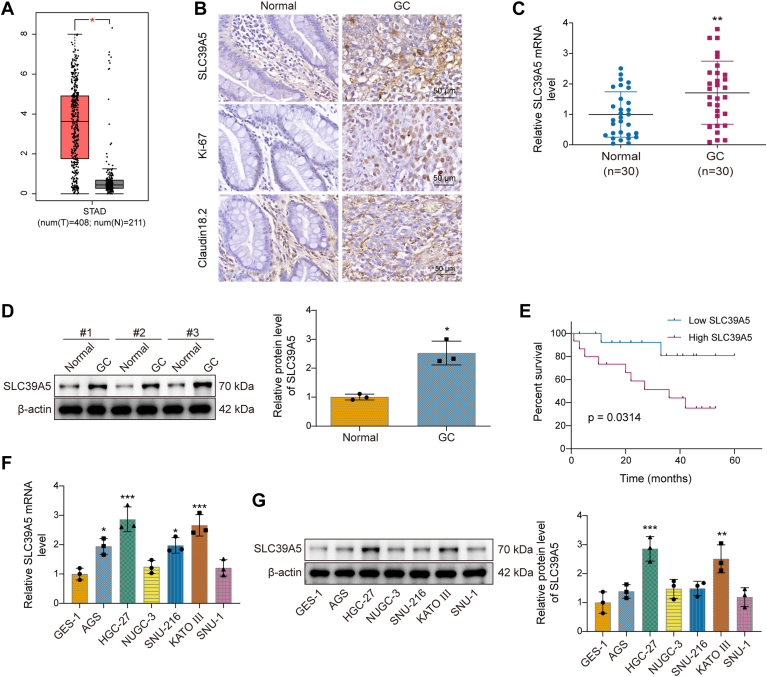
**SLC39A5 was upregulated in GC.***A*, SLC39A5 expression levels in GC tissues *versus* normal tissues were predicted by GEPIA. Thirty GC tissues and 30 adjacent tissues were collected from GC patients who had not received radiation or chemotherapy. *B*, SLC39A5, Ki67, and Claudin 18.2 were analyzed using IHC staining. The scale bar represents 50 μm. *C*, SLC39A5 mRNA levels in GC and adjacent tissues were measured by qRT–PCR, n = 30. *D*, SLC39A5 protein levels in GC and adjacent tissues were measured by Western blot, n = 3. *E*, prognostic analysis of SLC39A5 in GC, n = 30. The expression of SLC39A5 mRNA and protein expression in the human normal gastric epithelial cell line, GES-1, and GC cell lines, AGS, HGC-27, NUGC-3, SNU-216, KATO III, and SNU-1, was assessed by (*F*) qRT–PCR and (*G*) Western blot, n = 3. Data are expressed as mean ± SD. For *C*, an unpaired two-tailed Student's *t* test was performed to analyze data. For *D*, a paired two-tailed Student's *t* test was performed to analyze the data. For *F* and *G*, one-way ANOVA with Tukey's *post hoc* test was performed to analyze data. For *F* and *G*, one-way ANOVA with Tukey's *post hoc* test was performed to analyze data. ∗*p* < 0.05, ∗∗*p* < 0.01, and ∗∗∗*p* < 0.001. GC, gastric cancer; GEPIA, Gene Expression Profiling Interactive Analysis; IHC, immunohistochemistry; qRT–PCR, quantitative RT–PCR; SLC39A5, solute carrier family 39 member 5.

A previous study reported that SLC39A5 expression was upregulated in GC tissues (5). However, the specific functional role of SLC39A5 in GC remains unclear. We first analyzed public transcriptomic data using GEPIA, which predicted higher SLC39A5 expression in GC tissues compared with normal controls (Fig. 1*A*). We then collected tumor tissues and adjacent tissues from patients with GC. The results of immunohistochemistry (IHC) staining (Fig. 1*B*) revealed that the proliferation marker Ki-67 was highly expressed in GC tissues compared with adjacent normal tissues. To confirm the pathological identity of the GC tumor samples and validate tumor specificity, we also examined the expression of Claudin 18.2. Multiple studies have identified Claudin 18.2 as a promising diagnostic and therapeutic marker for GC (29, 30, 31), so we used it as a positive control to verify the identity of GC. As expected, both IHCs demonstrated significantly higher Claudin 18.2 expression in GC tissues compared with adjacent normal tissues, supporting the malignant origin of the samples (Fig. 1*B*). Next, we assessed SLC39A5 expression at both the transcript and protein levels. IHC staining (Fig. 1*B*) revealed markedly increased expression of SLC39A5 in GC tissues compared with adjacent normal tissues. Consistently, quantitative RT–PCR (qRT–PCR) and Western blot analyses (Fig. 1, *C* and *D*) demonstrated significantly increased mRNA and protein levels of SLC39A5 in GC tissues relative to normal healthy tissues. Moreover, elevated expression of SLC39A5 was positively correlated with the advanced tumor node metastasis stage in GC patients (Table 1). Furthermore, the upregulated SLC39A5 correlated with poor prognostic rate in GC patients (Fig. 1*E*). To further investigate the role of SLC39A5 in GC, we assessed its expression in several GC cell lines, including AGS, HGC-27, NUGC3, SNU216, KATO III, and SNU1. Compared with the normal human gastric epithelial cell line GES1, the mRNA expression of SLC39A5 was significantly upregulated in AGS, HGC-27, SNU216, and KATO III cells (Fig. 1*F*). Consistently, elevated protein levels of SLC39A5 were observed in HGC-27 and KATO III cells (Fig. 1*G*). Based on these results, HGC-27 and KATO III cell lines were selected for subsequent functional experiments. Altogether, high expression of SLC39A5 was associated with GC progression.

**Table 1:** Association between SLC39A5 expression and clinicopathological characteristics of GC patients

Clinical characteristics	SLC39A5			*P*
	N = 30	High (n = 15)	Low (n = 15)	
Gender				0.7152
Male	14	8	6	
Female	16	7	9	
Age (y)				0.7104
<55	12	5	7	
≥55	18	10	8	
TNM stage				
I–II	13	3	10	0.0253^a^
III–IV	17	12	5	
Tumor size (cm)				
<5	16	4	12	0.0092^b^
≥5	14	11	3	
Lymph node metastasis				0.2723
Negative	14	9	5	
Positive	16	6	10	
Differentiation				0.0253^a^
Good	13	10	3	
Poor	17	5	12	
Distant metastasis				0.0656
Yes	14	4	10	
No	16	11	5	

TNM, tumor node metastasis classification.

a *p* < 0.05.

b *p* < 0.01.

### SLC39A5 promoted GC cell proliferation, migration, and invasion

**Figure 2:**
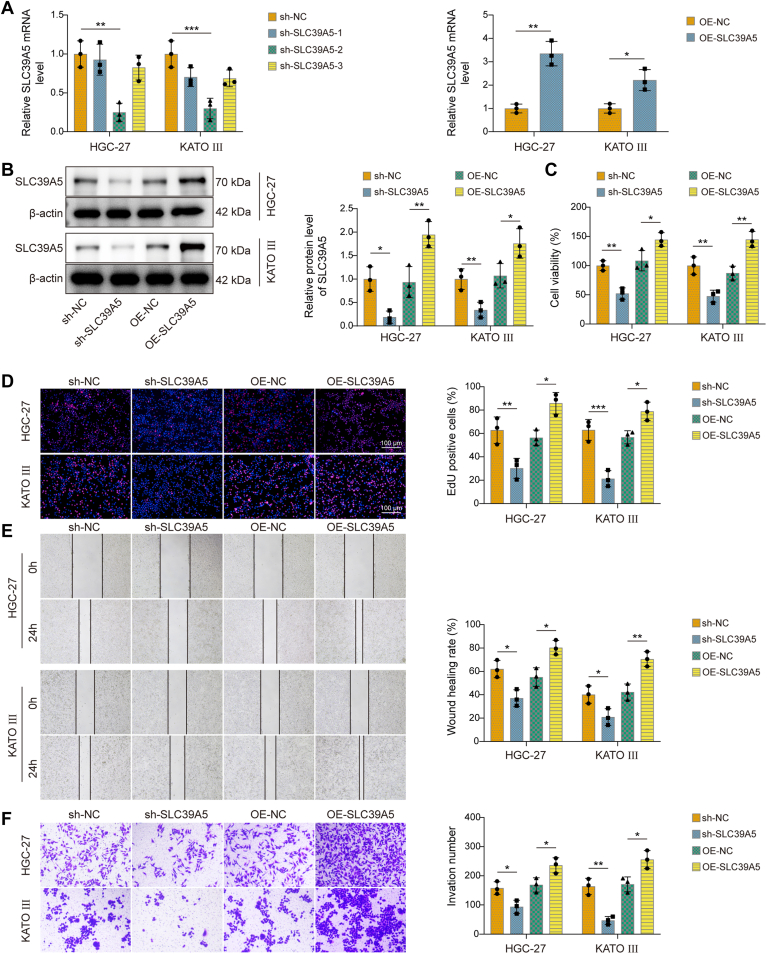
**Knockdown of SLC39A5 suppressed gastric cancer (GC) cell proliferation, migration, and invasion.** SLC39A5 was knocked down in HGC-27 and KATO III cells. *A*, SLC39A5 mRNA levels were measured in HGC-27 and KATO III cells by qRT–PCR. *B*, SLC39A5 protein levels were detected by Western blot in HGC-27 and KATO III cells. (*C*) Cell viability, (*D*) proliferation, (*E*) migration, and (*F*) invasion in HGC-27 and KATO III cells were assessed by CCK-8, EdU, Scratch, and Transwell assays, respectively. Data are expressed as mean ± SD, n = 3. For *A*, *C*–*F*, one-way ANOVA with Tukey's *post hoc* test was performed to analyze data. For *B*, an unpaired two-tailed Student's *t* test was performed to analyze data. ∗*p* < 0.05, ∗∗*p* < 0.01, and ∗∗∗*p* < 0.001. CCK-8, Cell Counting Kit-8; EdU, 5-ethynyl-2′-deoxyuridine; qRT–PCR, quantitative RT–PCR; SLC39A5, solute carrier family 39 member 5.

Next, we explored the functional role of SLC39A5 in GC by transfecting the HGC-27 and KATO III cells with sh-SLC39A5 or OE-SLC39A5. Three shRNA sequences (sh-SLC39A5-1, -2, and -3) were tested to determine the most effective knockdown. Among them, sh-SLC39A5-2 resulted in the most significant reduction in SLC39A5 expression, as confirmed by qRT–PCR and Western blot analyses (Fig. 2, *A* and *B*). Therefore, sh-SLC39A5-2 (abbreviated as sh-SLC39A5) was selected for all subsequent loss-of-function experiments. In parallel, successful upregulation of SLC39A5 following OE-SLC39A5 transfection was also verified. Functionally, SLC39A5 knockdown significantly suppressed GC cell proliferation, migration, and invasion (Fig. 2, *C*–*F*). In contrast, SLC39A5 overexpression (OE) showed the opposite result. Overall, SLC39A5 played a promoting role in the malignant phenotype of GC cells.

### SLC39A5 enhanced PIM1 enzymatic activity by increasing the influx of Zn^2+^

**Figure 3:**
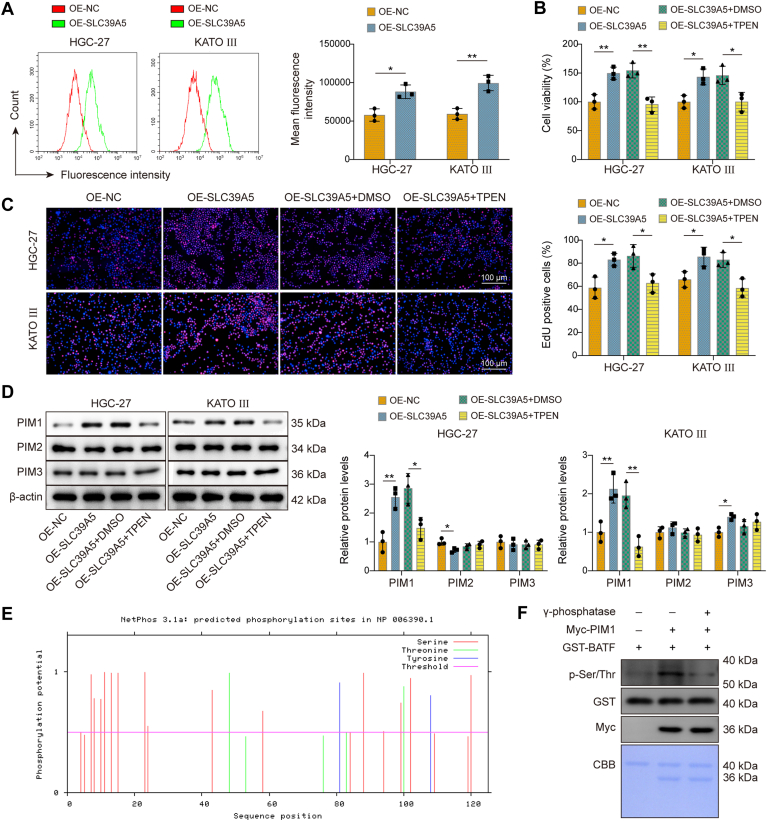
**SLC39A5 enhanced PIM1 enzymatic activity by increasing the influx of Zn^2+^.***A*, intracellular free Zn^2+^ levels in SLC39A5 overexpressed HGC-27 and KATO III cells were detected by flow cytometry. *B*–*D*, HGC-27 and KATO III cells were transfected with OE-NC or OE-SLC39A5 and then treated with DMSO or TPEN. *B*, cell proliferation was measured using the CCK-8 assay. *C*, cell proliferation was also measured using the EdU assay. *D*, PIM1/2/3 expression was detected by Western blot. *E*, phosphorylation sites in BATF were predicted using the Netphos 3.1 server. *F*, recombinant GST-BATF (expressed in *Escherichia coli*) and Myc-PIM1 (expressed in HEK-293T cells) were collected. The *in vitro* kinase assay was used to detect the phosphokinase activity of PIM1 in regulating BATF. Data are expressed as mean ± SD, n = 3. For *A*, an unpaired two-tailed Student's *t* test was performed to analyze data. For *B*–*D*, one-way ANOVA with Tukey's *post hoc* test was performed to analyze data. ∗*p* < 0.05, ∗∗*p* < 0.01. BATF, basic leucine zipper transcription factor; CCK-8, Cell Counting Kit-8; DMSO, dimethyl sulfoxide; EdU, 5-ethynyl-2′-deoxyuridine; GST, glutathione-*S*-transferase; HEK-293T, human embryonic kidney 293T cell line; NC, negative control; OE, overexpression; PIM1, proto-oncogene serine/threonine-protein kinase 1; SLC39A5, solute carrier family 39 member 5; TPEN, *N*,*N*,*N*′,*N*′-tetrakis(2-pyridylmethyl) ethylenediamine (zinc chelator); Zn, zinc.

SLC39A5 is known to function as a Zn transporter (32), which prompted us to further investigate whether it regulates GC progression by modulating Zn^2+^ influx. We first measured intracellular Zn^2+^ levels by flow cytometry and found that OE of SLC39A5 led to an increase in intracellular free Zn^2+^ influx (Fig. 3*A*). To further investigate the relationship between SLC39A5 and Zn, HGC-27 and KATO III cells were treated with the highly selective Zn^2+^ chelator, *N*,*N*,*N*′,*N*′-tetrakis (2-pyridylmethyl)ethylenediamine (TPEN). The results showed that the promotional effects of SLC39A5 OE on GC viability and proliferation were attenuated by TPEN treatment (Fig. 3, *B* and *C*). Zn homeostasis is known to play a critical role in regulating kinase activity (33). In this study, we aimed to investigate whether SLC39A5 modulates PIM kinase activity by regulating Zn^2+^ influx. It was found that OE of SLC39A5 resulted in increased PIM1 expression, whereas TPEN treatment reduced PIM1 expression (Fig. 3*D*). No significant changes were detected in the expression levels of PIM2 and PIM3 after SLC39A5 OE or TPEN treatment. These findings indicate that the SLC39A5–Zn axis primarily influences PIM1 among the PIM kinase family members in GC cells. As a serine/threonine protein kinase, PIM1 can regulate protein stability through its phosphorylation activity (34). This led us to investigate potential downstream substrates of PIM1. BATF, a member of the AP-1 transcription factor family, has been reported to exert oncogenic functions in GC (34, 35). Using the STRING database, we identified a potential protein–protein interaction between PIM1 and BATF among the PIM kinase family members (Fig. S1). Furthermore, NetPhos 3.1 prediction identified multiple phosphorylation sites on BATF (Fig. 3*E*), suggesting BATF may be regulated by PIM1 through phosphorylation. *In vitro* kinase assays showed that BATF protein was phosphorylated by PIM1, and the phosphokinase activity of PIM1 was suppressed by γ-phosphatase (enzymes that catalyze the dephosphorylation of proteins) (Fig. 3*F*). Taken together, these results suggested that SLC39A5 increased PIM1 enzymatic activity through mediating the influx of Zn^2+^.

### PIM1 stabilized BATF expression by phosphorylating it at serine 39 and threonine 381

**Figure 4:**
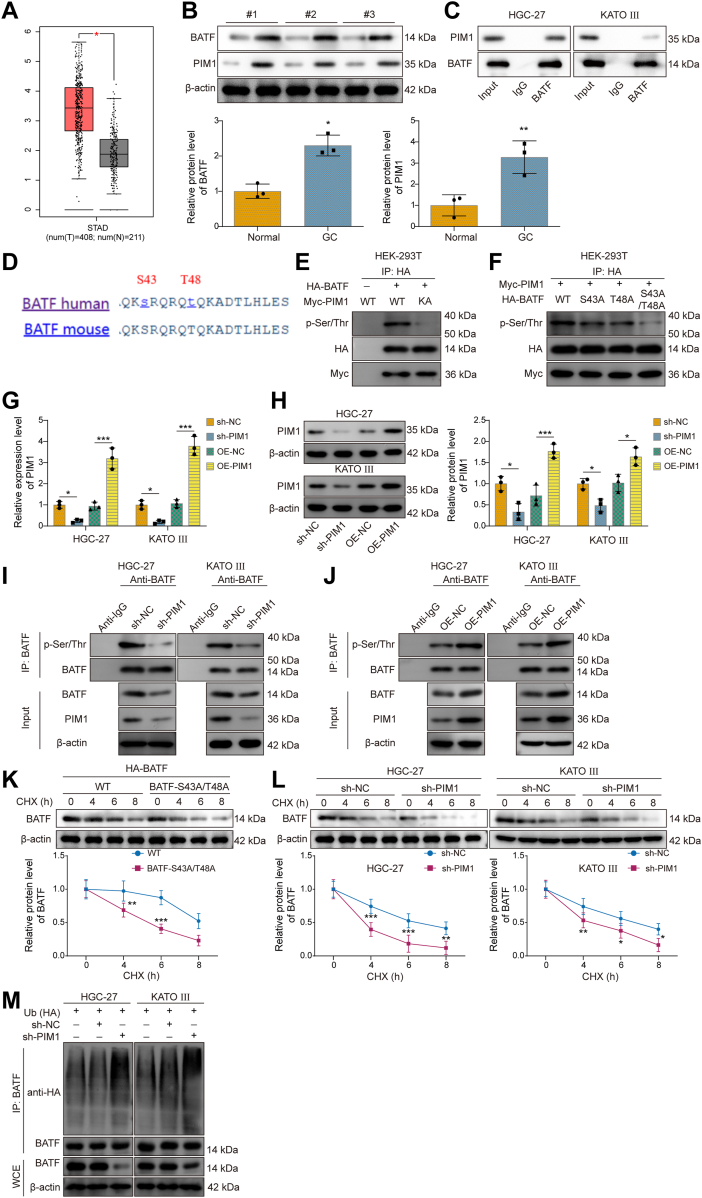
**PIM1 stabilized BATF expression by phosphorylating it at serine 39 and threonine 381.***A*, BATF expression in gastric cancer was predicted by the GEPIA (http://gepia.cancer-pku.cn/) database. *B*, BATF and PIM1 expression in gastric cancer tissues and adjacent noncancerous tissues were detected by Western blot. *C*, the interaction between BATF and PIM1 in HGC-27 and KATO III cells was analyzed by co-IP. *D*, conserved site analysis was performed for serine 43 (S43) and threonine 48 (T48) in BATF human and BATF mouse proteins. *E*, Myc-PIM1-WT, Myc-PIM1-KA, and HA-BATF were transfected in HEK-293T cells. Co-IP detected BATF phosphorylation levels in HEK-293T cells. *F*, HA-BATF (WT, S43A, T48A, or S43A/T48A) and Myc-PIM1 were transfected in HEK-293T cells. Co-IP revealed the phosphorylation of BATF in cells. The (*G*) mRNA and (*H*) protein expressions of PIM1 were detected in HGC-27 and KATO III cells transfected with sh-PIM1, OE-PIM1, or corresponding control vectors by qRT–PCR or Western blot, respectively. *I*, co-IP was performed to confirm phosphorylation of BATF in HGC-27 and KATO III cells cotransfected with sh-NC or sh-PIM1. *J*, co-IP was performed to confirm the phosphorylation of BATF in HGC-27 and KATO III cells cotransfected with OE-NC or OE-PIM1. *K*, BATF protein stability was assessed by Western blot after CHX treatment for 0, 4, 6, and 8 h in HA-BATF (WT or S43A/T48A) overexpressed HEK-293T cells. *L*, BATF protein stability was assessed by Western blot after CHX treatment for 0, 4, 6, and 8 h in HGC-27 and KATO III cells transfected with sh-NC or sh-PIM1. *M*, co-IP analysis of BATF ubiquitination in MHGC-27 and KATO III cells. Cells were cotransfected with HA-tagged ubiquitin (Ub-HA) and sh-NC/sh-PIM1. Ubiquitinated proteins were detected by immunoblotting with an anti-HA antibody. Whole-cell extracts (WCEs) were analyzed by Western blot to assess endogenous BATF. Data are expressed as mean ± SD, n = 3. For *B*, paired two-tailed Student's *t* test was performed to analyze data. For *G* and *H*, one-way ANOVA with Tukey's *post hoc* test was performed to analyze data. For *K* and *L*, two-way ANOVA with Sidak's multiple comparisons test was performed to analyze data. ∗*p* < 0.05, ∗∗*p* < 0.01, ∗∗∗*p* < 0.001. BATF, basic leucine zipper transcription factor; CHX, cycloheximide; Co-IP, coimmunoprecipitation; GEPIA, Gene Expression Profiling Interactive Analysis; HEK-293T, human embryonic kidney 293T cell line; OE, overexpression; PIM1, proto-oncogene serine/threonine-protein kinase 1; qRT–PCR, quantitative RT–PCR.

Subsequently, how PIM1 regulated BATF phosphorylation was investigated. GEPIA predicted that BATF was upregulated in GC (Fig. 4*A*). Western blot data further confirmed that both BATF and PIM1 were upregulated in GC tissues (Fig. 4*B*). The interaction between BATF and PIM1 in HGC-27 and KATO III cells was validated by coimmunoprecipitation (co-IP) (Fig. 4*C*). According to a previous report, BATF undergoes phosphorylation at serine 43 (S43) and threonine 48 (T48) (27), and we found these sites to be conserved in both humans and mice (Fig. 4*D*). To determine whether PIM1 affects the phosphorylation of BATF at S43 and T48, we overexpressed BATF and PIM1 in human embryonic kidney 293T (HEK-293T) cells. WT-PIM1 promoted BATF phosphorylation, whereas the kinase-inactive (KA) form of PIM1 (KA-PIM1) did not affect its phosphorylation (Fig. 4*E*). Site-directed mutagenesis further revealed that S43 and T48 were the specific phosphorylation sites where PIM1 acts on BATF (Fig. 4*F*). To examine the regulation of PIM1 and its impact on BATF in GC cells, HGC-27 and KATO III cells were transduced with sh-PIM1 or OE-PIM1 vectors. qRT–PCR and Western blot analyses confirmed that PIM1 expression was reduced by sh-PIM1, whereas increased by OE-PIM1 in both cell lines (Fig. 4, *G* and *H*). Co-IP assays demonstrated that PIM1 knockdown reduced BATF phosphorylation level in HGC-27 and KATO III cells, whereas PIM1 OE showed the opposite effect (Fig. 4, *I* and *J*). Moreover, mutation of BATF-S43/T48 or PIM1 knockdown led to reduced BATF protein stability (Fig. 4, *K* and *L*). Knockdown of PIM1 also increased BATF ubiquitination levels in both MHGC-27 and KATO III cells (Fig. 4*M*). These findings suggested that PIM1 stabilized BATF expression through phosphorylation at S43 and T48.

### SLC39A5 regulated GC cell proliferation, migration, and invasion through BATF

**Figure 5:**
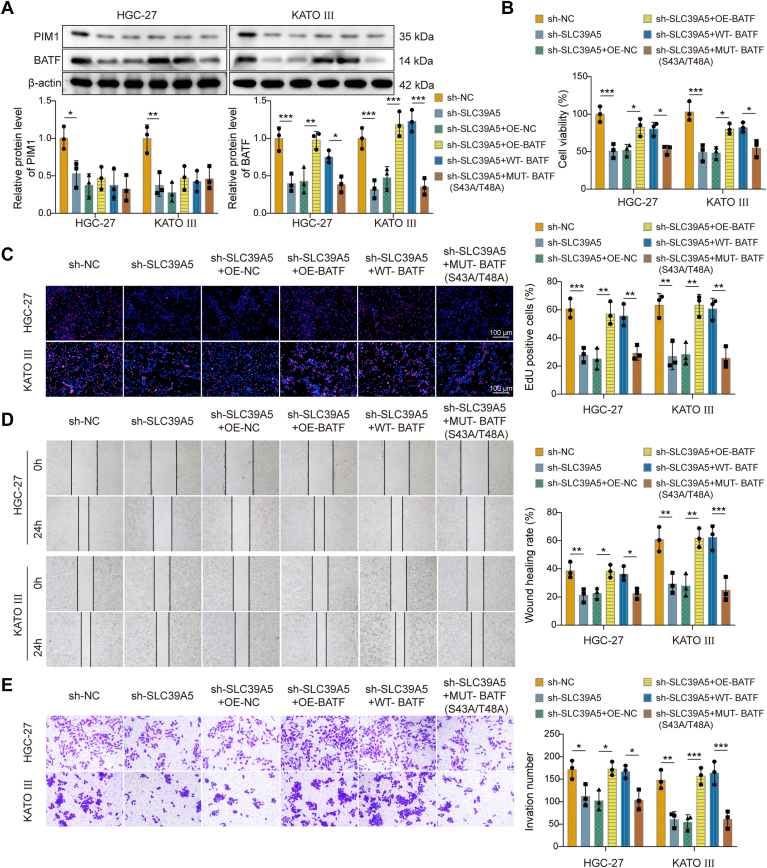
**SLC39A5 regulated gastric cancer cell proliferation, migration, and invasion through BATF.** HGC-27 and KATO III cells were transfected with sh-NC, sh-SLC39A5, sh-SLC39A5 + OE-NC, sh-SLC39A5 + OE-BATF, sh-SLC39A5 + WT-BATF, or sh-SLC39A5 + MUT-BATF (S43A/T48A). *A*, BATF and PIM1 protein levels were detected by Western blot in HGC-27 and KATO III cells. (*B*) Cell viability, (*C*) proliferation, (*D*) migration, and (*E*) invasion in HGC-27 and KATO III cells were assessed by CCK-8, EdU, Scratch, and Transwell assays, respectively. Data are expressed as mean ± SD, n = 3. One-way ANOVA with Tukey's *post hoc* test was performed to analyze data. ∗*p* < 0.05, ∗∗*p* < 0.01, and ∗∗∗*p* < 0.001. BATF, basic leucine zipper transcription factor; CCK-8, Cell Counting Kit-8; EdU, 5-ethynyl-2′-deoxyuridine; MUT, mutant; NC, negative control; OE, overexpression; PIM1, proto-oncogene serine/threonine-protein kinase 1; SLC39A5, solute carrier family 39 member 5.

We next examined whether SLC39A5 influences GC proliferation, migration, and invasion through BATF. Knockdown of SLC39A5 downregulated BATF expression (Fig. 5*A*). This effect was reversed by OE of WT-BATF but not by the BATF phosphorylation–deficient mutant (S43A/T48A). Furthermore, PIM1 protein levels were decreased following SLC39A5 knockdown; however, no significant change in PIM1 expression was observed upon BLTF OE or when cells were transfected with BATF containing S43A/T48A mutations (Fig. 5*A*). Moreover, BATF OE attenuated the inhibitory effects of SLC39A5 depletion on HGC-27 and KATO III cell viability, proliferation, migration, and invasion (Fig. 5, *B*–*E*). Nevertheless, the mutation of BATF at S43/T48 diminished the impact of BATF OE on GC cell behavior. These results implied that SLC39A5 regulated GC cell proliferation, migration, and invasion through the modulation of BATF expression and activity.

### BATF and JUN synergistically repressed Huntingtin-interacting protein 1–related expression and activated the PI3K–AKT pathway

**Figure 6:**
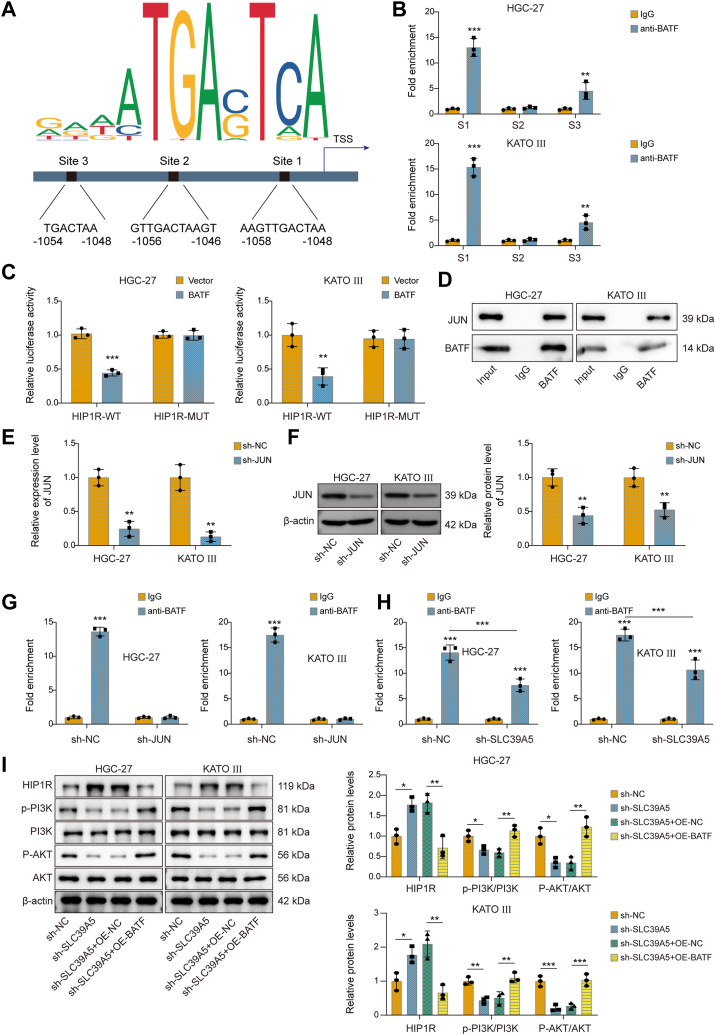
**BATF and JUN synergistically repressed HIP1R expression and activated the PI3K–AKT pathway.***A*, JASPAR predicted the conserved BATF–JUN composite DNA-binding motif within the HIP1R promoter region. *B*, the ChIP assay measured the specific binding sites of BATF to the HIP1R promoter region, at which BATF and JUN bind together. *C*, the dual-luciferase reporter assay tested the effect of BATF on the transcriptional activity of HIP1R. HIP1R-WT is a reporter gene vector containing the BATF–JUN conserved binding motif (S1 site), whereas HIP1R-MUT contains point mutations at the BATF–JUN binding motif (S1 site) to disrupt transcription factor binding. *D*, the interaction between BATF and JUN was verified by co-IP. *E*, qRT–PCR analysis of JUN mRNA expression in HGC-27 and KATO III cells transfected with sh-NC or sh-JUN. *F*, Western blot analysis of JUN protein levels in HGC-27 and KATO III cells following JUN knockdown. *G*, the regulatory relationship between BATF–JUN and HIP1R in JUN-depleted HGC-27 and KATO III cells was detected using a ChIP assay. *H*, the regulatory relationship between BATF–JUN and the HIP1R promoter in SLC39A5-depleted HGC-27 and KATO III cells was detected using a ChIP assay. *I*, the expression of HIP1R and PI3K–AKT pathway proteins, including p-PI3K, PI3K, p-AKT, and AKT, was detected by Western blot after SLC39A5 knockdown and/or BATF overexpression. Data are expressed as mean ± SD, n = 3. For *B*, *C*, *E*–*H*, an unpaired two-tailed Student's *t* test was performed to analyze data. For *I*, one-way ANOVA with Tukey's *post hoc* test was performed to analyze data. ∗*p* < 0.05, ∗∗*p* < 0.01, and ∗∗∗*p* < 0.001. AKT, protein kinase B; BATF, basic leucine zipper transcription factor; ChIP, chromatin immunoprecipitation assay; Co-IP, coimmunoprecipitation; HIP1R, Huntingtin-interacting protein 1–related; JUN, Jun proto-oncogene AP1 transcription factor subunit; MUT, mutant; NC, negative control; qRT–PCR, quantitative RT–PCR; SLC39A5, solute carrier family 39 member 5.

The BATF family commonly forms transcriptional repression complexes with JUN proteins (36). JASPAR database predicted that the Huntingtin-interacting protein 1–related (HIP1R) promoter region contains conserved DNA-binding motifs recognized by both BATF and JUN (called BATF–JUN complex motif). Figure 6*A* showed the three most conservative sites, namely site 1 (S1), site 2 (S2), and site 3 (S3). Chromatin immunoprecipitation (ChIP) results indicated that S1 was the regulatory site for BATF–JUN–HIP1R promoter interaction, which was further confirmed by dual-luciferase assays (Fig. 6, *B* and *C*). What is more, co-IP experiments demonstrated an interaction between BATF and JUN (Fig. 6*D*). To evaluate the role of JUN in this regulatory axis, JUN expression was knocked down in HGC-27 and KATO III cells using sh-JUN. qRT–PCR and Western blot analyses confirmed effective silencing of JUN at both the mRNA and protein levels (Fig. 6, *E* and *F*). Notably, the downregulation of JUN decreased the BATF and HIP1R promoter interaction (Fig. 6*G*), suggesting that BATF and JUN form a complex that influences HIP1R transcription. Furthermore, the knockdown of SLC39A5 also significantly decreased the enrichment of HIP1R in BATF-immunoprecipitated fractions (Fig. 6*H*). Previous studies have shown that HIP1R is involved in the regulation of the PI3K–AKT signaling pathway, which plays a critical role in cancer cell proliferation and survival (36). To explore whether the SLC39A5–BATF–HIP1R axis affects this pathway, we examined the levels of phosphorylated PI3K (p-PI3K) and p-AKT following SLC39A5 depletion. Depletion of SLC39A5 upregulated HIP1R expression and decreased the levels of p-PI3K and p-AKT, which was reversed by the OE of BATF (Fig. 6*I*). In summary, BATF and JUN synergistically inhibited HIP1R expression and activated the PI3K–AKT pathway.

### SLC39A5 regulated GC cell proliferation, migration, and invasion by suppressing HIP1R

**Figure 7:**
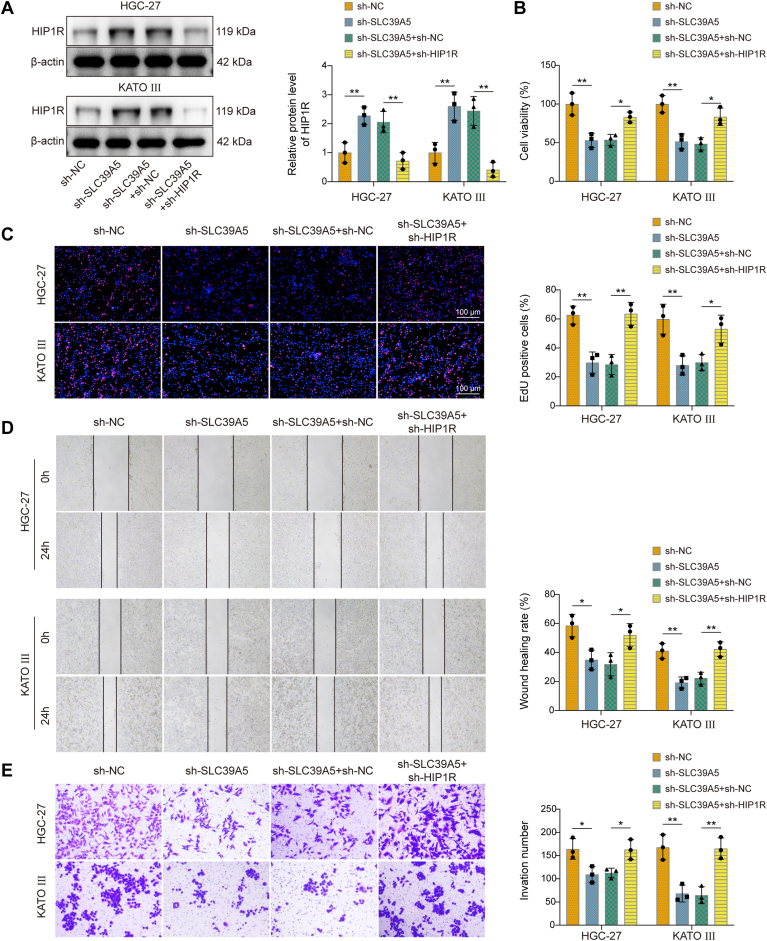
**SLC39A5 suppressed HIP1R to regulate gastric cancer cell proliferation, migration, and invasion.** HGC-27 and KATO III cells were transfected with sh-NC, sh-BATF, sh-SLC39A5 + sh-NC, sh-SLC39A5 + sh-HIP1R. *A*, HIP1R protein levels were detected by Western blot in HGC-27 and KATO III cells. (*B*) Cell viability, (*C*) proliferation, (*D*) migration, and (*E*) invasion in HGC-27 and KATO III cells were assessed by CCK-8, EdU, Scratch, and Transwell assays, respectively. Data are expressed as mean ± SD, n = 3. One-way ANOVA with Tukey's *post hoc* test was performed to analyze data. ∗*p* < 0.05, ∗∗*p* < 0.01. BATF, basic leucine zipper transcription factor; CCK-8, Cell Counting Kit-8; EdU, 5-ethynyl-2′-deoxyuridine; HIP1R, Huntingtin-interacting protein 1–related; NC, negative control; SLC39A5, solute carrier family 39 member 5.

We next investigated whether SLC39A5 affects GC proliferation, migration, and invasion through HIP1R. Knockdown of SLC39A5 led to upregulation of HIP1R expression, whereas transfection with sh-HIP1R resulted in downregulation of HIP1R expression (Fig. 7*A*). Silencing of HIP1R diminished the inhibitory effect of SLC39A5 knockdown on GC cell viability, proliferation, migration, and invasion (Fig. 7, *B*–*E*). Taken together, SLC39A5 promoted GC cell proliferation, migration, and invasion *via* HIP1R.

### SLC39A5 promoted gastric tumor growth through the BATF–HIP1R–PI3K–AKT axis

**Figure 8:**
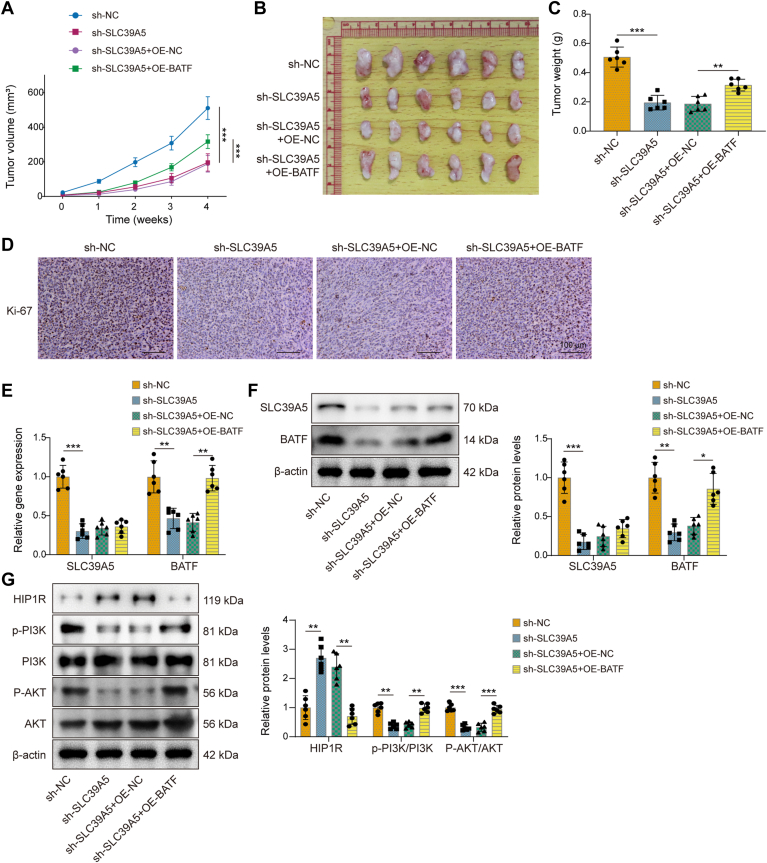
**SLC39A5 promoted gastric tumor growth through BATF–HIP1R–PI3K–AKT axis.** A total of 24 mice were divided into four groups. Each group received subcutaneous injections of HGC-27 cells (0.5 × 10^6^ cells/kg) transfected with sh-NC, sh-SLC39A5, sh-SLC39A5 + OE-NC, or sh-SLC39A5 + OE-BATF. *A*, the tumor volume in nude mice of each group. *B*, images of the tumors. *C*, the tumor weight in nude mice of each group. *D*, IHC detected the expression of Ki-67 in GC tumor tissues. The scale bar represents 100 μm. The mRNA and protein levels of SLC39A5 and BATF in GC tumor tissues were detected by (*E*) qRT–PCR and (*F*) Western blot. *G*, the protein expression of HIP1R and PI3K–AKT pathway proteins, including p-PI3K, PI3K, p-AKT, and AKT, was assessed by Western blot. Data are expressed as mean ± SD, n = 6. For *C*, *E*–*G*, one-way ANOVA with Tukey's *post hoc* test was performed to analyze data. ∗*p* < 0.05, ∗∗*p* < 0.01, and ∗∗∗*p* < 0.001. AKT, protein kinase B; BATF, basic leucine zipper transcription factor; GC, gastric cancer; HIP1R, Huntingtin-interacting protein 1–related; IHC, immunohistochemistry; NC, negative control; OE, overexpression; qRT–PCR, quantitative RT–PCR; SLC39A5, solute carrier family 39 member 5.

The roles of SLC39A5 and BATF in GC were then validated *in vivo* using xenograft models. The knockdown of SLC39A5 alone inhibited tumor growth of nude mice, whereas OE of BATF promoted tumor growth, mitigating the effects of SLC39A5 knockdown (Fig. 8, *A*–*C*). Subsequent analysis showed that Ki-67 levels decreased following SLC39A5 knockdown, whereas Ki-67 levels increased with BATF OE (Fig. 8*D*). Injection of sh-SLC39A5 resulted in decreased expression of both SLC39A5 and BATF (Fig. 8, *E* and *F*). In contrast, injection of OE-BATF led to an increase in BATF expression, whereas the expression of SLC39A5 remained unchanged. In addition, the silencing of SLC39A5 led to the upregulation of HIP1R expression and the downregulation of p-PI3K and p-AKT levels, which were reversed by OE of BATF (Fig. 8*G*).

To address the limitations of subcutaneous xenograft models and better reflect the pathological significance of GC, we also established an orthotopic tumor model in nude mice. Tumor fragments derived from subcutaneous xenografts established using HGC-27 cells with SLC39A5 knockdown alone or in combination with BATF OE. These tumor fragments were surgically placed into the serosal surface of the greater curvature of the stomach (Fig. S2*A*). Throughout the experiment, there were no significant differences in body weight across groups, suggesting that the interventions did not cause systemic toxicity (Fig. S2*B*). Gross examination of the stomachs revealed markedly smaller orthotopic tumors in the sh-SLC39A5 group compared with controls, whereas BATF OE partially rescued tumor growth (Fig. S2*C*). This was further confirmed by tumor weight measurements (Fig. S2*D*). Western blot analysis showed that SLC39A5 knockdown led to reduced expression of both SLC39A5 and BATF, whereas BATF expression was restored upon OE-BATF, with SLC39A5 levels remaining unchanged (Fig. S2*E*). In parallel, SLC39A5 knockdown increased HIP1R expression and reduced phosphorylation of PI3K and AKT; these effects were reversed by BATF OE (Fig. S2*F*), supporting the involvement of a BATF–HIP1R–PI3K–AKT signaling axis. Importantly, H&E staining of liver tissues demonstrated that SLC39A5 depletion significantly suppressed GC liver metastasis, which was abolished upon BATF OE (Fig. S2*G*). These results suggested that SLC39A5 enhanced the GC tumor growth *via* regulating the BATF–HIP1R–PI3K–AKT axis.

## Discussion

Despite advances in diagnostic and therapeutic approaches to GC, early detection remains a challenge, resulting in late-stage diagnoses and poor survival outcomes (37). Previous studies have demonstrated that SLC39A5 was involved in regulating GC and may serve as a potential prognostic biomarker (5). However, the exact role of SLC39A5 and its underlying mechanisms remain to be explored. Ultimately, we confirmed that SLC39A5 contributed to GC progression *via* the BATF–HIP1R axis. This study provides new insights into using SLC39A5 as a potential diagnostic target for treating GC.

Emerging evidence indicates a compelling association between Zn deficiency and cancer (38). In *in vivo* studies, it has been demonstrated that treatment with Zn enhanced resistance to tumors in mice (39) and reduced the occurrence of spontaneous tumors in mice (40). Furthermore, Zn deficiency has been linked to larger tumor size, more advanced tumor stages, and a higher frequency of unplanned hospitalizations in head and neck cancer patients (41). In a prospective cohort study, higher dietary Zn intake was associated with a reduced risk of developing colon cancer (42). SLC39A5, a member of the leucine zipper (ZIP) family of Zn transporters, plays a crucial role in regulating intracellular Zn levels by transporting Zn into cells from the extracellular environment (43). Previous studies have documented that SLC39A5 is key in modulating these vital biological processes and various diseases, including several cancers (44). For example, SLC39A5 was elevated in human esophageal cancer, and its depletion inhibited esophageal cancer cell growth (7). It has also been shown that SLC39A5-induced lung adenocarcinoma cell proliferation suppresses apoptosis *via* the PI3K–AKT signaling pathways (7). Furthermore, SLC39A5 was abnormally expressed in GC (5), suggesting its involvement in GC progression as well. Consistently, we found that SLC39A5 expression was elevated in GC and strongly correlated with a reduced survival rate in patients. In addition, we exhibited that the knockdown of SLC39A5 attenuated GC cell proliferation, migration, and invasion *in vitro* well as GC tumor growth *in vivo*. These findings indicated that SLC39A5 may play a critical role in mediating the progression of GC.

Zn can modulate the activity of numerous enzymes, including kinases and phosphatases, and plays a critical role in intracellular signaling regulation (45). Dysregulated Zn homeostasis has been increasingly recognized as a key factor affecting kinase-mediated processes critical for cancer progression (46). For example, in gastric adenocarcinoma, upregulation of SLC39A10 enhanced intracellular Zn^2+^ levels, thereby activating casein kinase 2 and its downstream mitogen-activated protein kinase/extracellular signal–regulated kinase and PI3K/AKT pathways, ultimately promoting tumor proliferation and progression (46). Zn deficiency in GC patients was associated with poor prognosis and may promote tumor progression by disrupting PI3K–AKT, NF-κB, and Wnt/β-catenin signaling pathways and impairing immune-related kinase functions (47). As a member of the SLC39A Zn transporter family, SLC39A5 has also been implicated in regulating Zn homeostasis and thereby modulating kinase-driven cellular functions. For instance, Wang *et al.* (10, 32) demonstrated that SLC39A5 governed glucose sensing and insulin secretion in pancreatic β-cells through sirtuin 1 and peroxisome proliferator–activated receptor gamma coactivator 1-alpha–mediated regulation of glucose transporter type 2, further supporting the concept that Zn transporters are closely linked to kinase signaling cascades. Our findings indicate that SLC39A5 OE and Zn^2+^ chelation predominantly regulated PIM1 expression, with minimal effects on PIM2 and PIM3. PIM1 is a serine/threonine kinase known to act as an oncogene in multiple cancers, such as leukemia, myeloma, breast and prostate cancers, by phosphorylating target proteins involved in apoptosis, cell cycle progression, migration, and metabolism (48). It was shown that PIM1 phosphorylated N-myc downstream–regulated gene 1 to mediate prostate cancer cell invasion and metastasis (49). Furthermore, PIM1 phosphorylated P-glycoprotein, shielding it from proteasomal degradation, which augmented its expression on the cell surface and contributed to breast cancer resistance to doxorubicin (50). Although the exact mechanism by which SLC39A5 regulates PIM1 in GC remains unclear, existing evidence suggests possible pathways. Zn finger E-box binding homeobox 1 (ZEB1), a Zn^2^-dependent transcription factor, has been shown to promote GC progression (51, 52), indicating that SLC39A5 may activate ZEB1 *via* Zn^2+^ influx. Moreover, PIM1 is regulated by various miRs (53, 54, 55), and Zn^2+^ has been implicated in modulating miR expression (56, 57). Of note, miR-33a suppresses GC by targeting PIM1 (53), and ZEB1 can repress miR-33a-5p (58). These findings suggest a potential regulatory pathway in which SLC39A5 promotes PIM1 expression through Zn^2+^-mediated activation of ZEB1 and suppression of miR-33a-5p. This mechanism may explain the selective upregulation of PIM1 by SLC39A5. The specificity of this regulatory axis merits further investigation in future studies.

BATF is a member of the AP-1/ATF superfamily of transcription factors (22). Functionally, BATF serves as a negative regulator of AP-1-dependent transcription and is essential for the differentiation and function of various immune cell types (59). Recent studies have elucidated post-translational regulatory mechanisms affecting BATF activity, particularly phosphorylation (22). For example, S43 and T48 within its DNA-binding domain have been identified as phosphorylation sites targeted by PKC in Jurkat T cells (27). However, the full spectrum of kinases involved and the functional consequences of BATF phosphorylation remain incompletely characterized. In this study, we demonstrate that PIM1 directly phosphorylates BATF at S43 and T48, thereby enhancing its protein stability. Our study mainly verified that SLC39A5, as a Zn^2+^ transporter, promoted the expression of protein kinase PIM1 in GC. The upregulated PIM1 further enhanced the phosphorylation and protein stability of BATF through its kinase activity. Whether SLC39A5, as a Zn^2+^ transporter, can directly regulate BATF expression independently of the PIM1-mediated phosphorylation pathway remains to be clarified.

BATF contains a ZIP domain that enables dimerization, typically forming heterodimers with Jun family proteins, such as c-Jun, JunD, and JunB (60). These heterodimers bind to AP-1 consensus DNA sequences *via* the basic region adjacent to the ZIP motif, thereby functioning as negative regulators of AP-1 activity (61, 62, 63). The BATF–JUN complex has been shown to modulate a variety of downstream genes. For example, this complex could transcriptionally inhibit Sirt1 expression in T cells (22), induce matrix-degrading enzymes, and downregulate cartilage matrix molecules in chondrocytes (36). Building on these findings, we identified that BATF formed a complex with JUN to bind the AP-1 consensus site within the HIP1R promoter. After knocking down JUN, BATF lost its heterodimerization partner, leading to impaired stabilization on the AP-1 motif and a significant reduction in its binding to the HIP1R promoter. HIP1R, a protein involved in endocytosis and cellular trafficking, has been reported as a tumor inhibitor in various types of cells. In GC cancer, HIP1R has been shown to inhibit tumor progression (64). In addition, Zhu *et al.* (65)demonstrated that HIP1R repression, mediated by DNA methylation and miR-92a-3p, promoted pancreatic cancer progression through activation of the PI3K–AKT signaling pathway. Consistent with these findings, our study not only confirmed that transcriptional suppression of HIP1R activates the PI3K–AKT pathway in GC but also revealed that the BATF–JUN complex directly contributes to this process by synergistically repressing HIP1R expression.

In conclusion, our research elucidates a regulatory axis involving SLC39A5, PIM1, BATF, and HIP1R, contributing to GC progression. We demonstrate that SLC39A5 enhances PIM1 activity by increasing Zn influx, leading to the phosphorylation and stabilization of BATF. The stabilized BATF forms a complex with JUN, which represses HIP1R expression. This repression subsequently activates the PI3K–AKT pathway, promoting malignant behaviors in GC cells. These results shed light on the molecular mechanisms driving GC and highlight potential therapeutic targets for future interventions.

While our study provides significant insights into the role of SLC39A5 in GC progression, certain limitations must be acknowledged. The SLC39 family consists of more than 10 members, and the roles of other family members have not been explored. In addition, the upstream regulatory mechanisms of SLC39A5 and how SLC39A5 affects PIM1 were not investigated because of funding restrictions.

## Experimental procedures

### Clinical sample collection

Thirty GC tissues and 30 adjacent healthy gastric tissues were collected from GC patients who had not undergone radiation or chemotherapy. The samples were spontaneously frozen in liquid nitrogen after collection. All research protocols involving human samples were approved by the Research Ethics Review Committee of The Second Hospital of Jilin University, and informed consent was obtained from each participant. The correlation of SLC39A5 expression and clinical characteristics is shown in Table 1. The studies abide by the Declaration of Helsinki principles.

### IHC staining

GC tumor tissues were preserved in 3.7% buffered formalin before being embedded in paraffin. Sections measuring 5 μm were then cut from these paraffin blocks and subjected to IHC staining. The sections were incubated with the primary antibody anti-SLC39A5 (MA527260, 1:500 dilution; Thermo Fisher Scientific), anti-Ki-67 (ab16667, 1:200 dilution; Abcam), or anti-Claudin 18.2 (ab241330, 1:600 dilution; Abcam) at room temperature for 1 h. After primary antibody incubation, secondary antibodies were applied. The sections were then counterstained with hematoxylin and imaged using the SP8 UV/Visible Laser Confocal Microscope (Leica).

### Cell culture and transfection

The GC cell lines, AGS, HGC-27, NUGC3, SNU216, KATO III, and SNU1, as well as the human gastric epithelial cell line GES1, were sourced from the American Type Culture Collection. All cell lines used in this study were authenticated using short tandem repeat profiling and tested negative for mycoplasma contamination *via* PCR and culture assays. All cell lines were cultured in RPMI1640 medium (Gibco), which was enriched with 10% fetal bovine serum (Gibco), 100 units/ml of penicillin, and 100 μg/ml of streptomycin.

GC cells were seeded at a density of 5 × 10^6^ cells per well 1 day before transfection. shRNAs targeting SLC39A5 (sh-SLC39A5-1-3), PIM1 (sh-PIM1), JUN (sh-JUN), HIP1R (sh-HIP1R), and a control shRNA were incorporated into GV102 vectors. For OE studies, the full-length SLC39A5, PIM1, or BATF gene sequences were amplified by PCR and cloned into the pcDNA3.1 vector (Promega) to create OE-SLC39A5, OE-PIM1, or OE-BATF plasmids, respectively. Transfections were performed using Lipofectamine 3000 (Invitrogen) according to the manufacturer’s instructions. The cells were incubated for 48 h before being subjected to *in vitro* analyses. The shRNA sequences are shown in Table 2.

**Table 2:** The shRNA sequences

shRNA	Sequence
sh-SLC39A5-1	TCATTGGCTGACCACCTGAAT
sh-SLC39A5-2	GGTGACCTGGAAGAGTCAAAG
sh-SLC39A5-3	TTTGGCCTGTACGGCGAGAAT
sh-PIM1	ACGTGGAGAAGGACCGGATTT
sh-JUN	ACTCATGCTAACGCAGCAGTT
sh-HIP1R	CAGAAGGCCCTGGTGGATAAT

Lentiviral particles were produced in HEK-293T cells using a third-generation packaging system. Briefly, 293T cells were seeded in 10-cm dishes and cultured until they reached 70 to 80% confluency. Cells were then cotransfected with 2 μg of the expression plasmid (sh-NC, OE-NC, sh-SLC39A5, or OE-BATF), 1.5 μg of psPAX2, and 0.5 μg of pMD2.G using polyethylenimine in Opti-MEM (Thermo Fisher Scientific). After 6 h, the medium was replaced with fresh complete Dulbecco's modified Eagle's medium. Viral supernatants were collected at 72 h post-transfection, filtered through a 0.45 μm membrane to remove cell debris, and stored at 4 °C for immediate use or concentrated and frozen at −80 °C for later use. HGC-27 cells were seeded in 6-well plates to reach 30% to 50% confluency at the time of infection. Filtered lentiviral supernatants were used to infect the cells in the presence of 8 μg/ml polybrene. After 48 h, the medium was replaced, and cells were selected with puromycin (2 μg/ml) for 12 days to establish stable cell lines. The resulting stably transduced cells were expanded in complete medium for animal experiments.

### Cell treatment

HGC-27 and KATO III cells were seeded in 15 cm culture dishes (Corning, Inc) at a density of 5 × 10^5^ cells/ml. The cells were treated with 3.5 μM TPEN (616394100MG; Merck Millipore), a cell-permeant metal ion chelator known to have high affinities with Zn and being used for limiting the available Zn, for 24 h or 100 μg/ml cycloheximide (Sigma–Aldrich) for 0, 4, 6, and 8 h. After the treatments, the cells were collected for subsequent Western blot analysis.

### Cell Counting Kit-8 assay

To evaluate cell proliferation, a Cell Counting Kit-8 assay (ab228554; Abcam) was utilized. Cells were distributed into 96-well plates at a concentration of 5000 cells per well. After a 48-h incubation period, 10 μl of Cell Counting Kit-8 solution was introduced to each well. The plates were then incubated for an additional 4 h, after which the absorbance was recorded using a microplate reader (Thermo Fisher Scientific).

### 5-Ethynyl-2′-deoxyuridine proliferation assay

The proliferation of cells was examined by the 5-ethynyl-2′-deoxyuridine (EdU) Assay/EdU Staining Proliferation Kit (ab219801; Abcam) following the manufacturer’s protocol. Briefly, 50,000 cultured GC cells were seeded into a 4-well plate and incubated overnight. The cells were treated with 10 μM EdU for 10 h, then fixed, washed, and permeabilized. A Click-iT reaction cocktail was added for half an hour, followed by Hoechst 33342 staining for another 30 min. The images were captured using the SP8 UV–Visible Laser Confocal Microscope.

### Scratch assay

Cells were cultured in 6-well plates at a density of 5 × 10^4^ cells/cm^2^ in F/12K medium for 24 h to achieve a confluent monolayer. A wound was then created by scratching the cell monolayer with a 200 μl pipette tip. After removing the medium, the cells were washed twice with PBS and then incubated in a serum-free medium. Wound closure was observed using a DMi8 optical microscope (Leica). Images taken at the start and end of the 48 h were used to assess wound healing.

### Transwell assay

Matrigel (BD Biosciences) was utilized to coat the membranes of Transwell chambers, which were then placed into wells of a 24-well plate. Each Transwell chamber received 500 μl of serum-free RPMI1640 medium containing 5 × 10^3^ cells. In the lower wells, 750 μl of RPMI1640 medium supplemented with 10% fetal bovine serum was added to act as a chemoattractant. The plates were incubated for 48 h to allow cell invasion. The invasive cells on the lower surface were stained with crystal violet for 5 min. The stained cells were visualized and imaged using the DMi8 optical microscope.

### Measurement of intracellular Zn^2+^

The intracellular free Zn^2+^ concentration was assessed using the Zn^2+^ probe FluoZin3 (Molecular Probes, Invitrogen) *via* flow cytometry (Leica) based on a previously published protocol (66). For the analysis, the specified cells were subjected to trypsinization, centrifugation, and washing. Subsequently, the cells were incubated with 1 μM FluoZin3 for 50 min at 37 °C. Following a 30-min incubation, the fluorescence intensity was measured at an emission wavelength of 516 nm and an excitation wavelength of 494 nm.

### Co-IP assay

To examine protein interactions and the ubiquitination level of BATF, co-IP was performed. The cells were first washed with PBS and then treated with IP lysis buffer (Merck Millipore). The treated cells were sonicated on ice and subsequently centrifuged at 12,000 rpm for 10 min. From the resulting supernatant, 30 μl was set aside as the input sample. The remaining 420 μl of the supernatant was incubated overnight at 4 °C with either an anti-BATF antibody (ab236876, 1:30 dilution; Abcam) or a nonspecific immunoglobulin G (ab172730, 1:1000 dilution; Abcam). After incubation, protein A was introduced to the samples, followed by an additional hour of incubation at 4 °C. The samples were washed four times with IP buffer, followed by a 2-min centrifugation and discarding of the supernatant. Finally, 30 μl of 2× SDS lysate was added to each sample and incubated for 10 min before performing immunoblotting with the anti-PIM1 antibody (ab300453, 1:1000 dilution; Abcam), anti-JUN (MA5-15172, 1:1000 dilution; Thermo Fisher Scientific) or anti-HA tag antibody (ab236632, 1:1000 dilution; Abcam).

To assess BATF phosphorylation levels, we obtained the full-length complementary DNA (cDNA) ORF sequences for BATF and PIM1 from Origene. We generated mutants through PCR-based site-directed mutagenesis using the QuikChange Site-Directed Mutagenesis Kit (Stratagene). The mutants included BATF serine-to-alanine substitution at position 43 (BATF-S43A), BATF threonine-to-alanine substitution at position 48 (BATF-T48A), double mutant at both positions (BATF-S43A/T48A), and kinase-inactive PIM1 mutant (PIM1-KA). All these constructs were subcloned into pcDNA3.1 vectors using primers that incorporated HA-tags or Myc-tags at their C-terminal ends. HEK-293T cells were transfected with these plasmids (including HA-BATF, HA-BATF-S43A, HA-BATF-T48A, HA-BATF-S43A/T48A, Myc-PIM1, and Myc-PIM1-KA). The supernatants were collected, and nonspecific proteins were removed by incubating with protein G-plus beads (Santa Cruz Biotechnology) for 30 min at 4 °C. The mixture was then centrifuged at 1000*g* for 5 min at 4 °C. The cleared supernatant was incubated with either an anti-HA tag antibody (ab236632, 1:30 dilution; Abcam) or an anti-Myc tag antibody (ab32, 1:50 dilution; Abcam) for 1 h at 4 °C on a rotating platform. Protein G-plus beads were added to the samples, and incubation continued overnight at 4 °C with rotation. The following day, the beads were washed four times with ice-cold 0.2% digitonin wash buffer (150 mM NaCl, 50 mM Tris, pH 7.4) and twice with ice-cold PBS. For immunoblot analysis, proteins were eluted from the beads by boiling them and analyzed by Western blot.

### *In vitro* kinase assay

To investigate the kinase activity of PIM1 in regulating BATF phosphorylation, *in vitro* kinase assay was conducted (67). The glutathione S-transferase-BATF fusion protein was expressed in *Escherichia coli* BL21(DE3), induced with 0.5 mM IPTG (34060; Thermo Fisher Scientific) at 16 °C overnight, and purified from bacterial lysates using Glutathione Sepharose 4B affinity chromatography with elution in 10 mM reduced glutathione. Myc-tagged PIM1 was induced in HEK-293T cells, followed by IP from cell lysates using anti-Myc agarose beads. Then the recombinant glutathione-S-transferase-BATF and Myc-tagged PIM1 were incubated in a 40 μl kinase assay buffer containing 20 mM Tris–HCl (pH 7.4), 20 mM NaCl, 10 mM MgCl_2_, 1 mM DTT, and 50 μM ATP (GE Healthcare) at 30 °C for 30 min. γ-phosphatase was added as a control sample to verify kinase dependence. The reaction mixtures were resolved by SDS-PAGE and transferred to polyvinylidene fluoride membranes. The phosphorylation was detected by immunoblotting with anti–phospho-serine/threonine antibody (ab17464, 1:1000 dilution; Abcam). Proteins were examined by Coomassie blue staining prior to use in kinase reactions.

### ChIP assay

Cells were harvested and lysed using IP lysis buffer (Merck Millipore). The lysates underwent crosslinking and sonication. Next, the lysates were combined with 900 μl of ChIP Dilution Buffer, 20 μl of 50× Protease Inhibitor Cocktail, and 60 μl of Protein A Agarose/Salmon Sperm DNA beads. After incubation, the lysates were centrifuged to obtain the supernatant, which was carefully transferred to a new tube. To the supernatant, 1 μl of either anti-BATF antibody (ab236876, 1:500 dilution; Abcam) or anti–immunoglobulin G antibody (ab205718, 1:1000 dilution; Abcam) was added, and the mixture was incubated overnight at 4 °C for IP. Following the overnight incubation, samples were washed and treated with 1 μl of RNase A, followed by a 37 °C incubation for 1 h to degrade RNA. Subsequently, 10 μl of 0.5 M EDTA, 20 μl of 1 M Tris–HCl, and 2 μl of 10 mg/ml proteinase K were added to the samples, which were then incubated at 45 °C for 2 h. Finally, DNA was extracted from the samples and quantified using qPCR.

### Dual-luciferase reporter assay

The JASPAR database predicted the common conserved DNA-binding motif that BATF and JUN bind to the HIP1R promoter. This common conserved binding motif was named BATF–JUN. Next, the WT promoter region of the HIP1R gene (HIP1R-WT), containing the predicted BATF–JUN binding motif, was amplified from human genomic DNA and cloned into the psiCHECK2 vector (Promega). The mutant construct (HIP1R-MUT) was generated by introducing point mutations into the binding site to disrupt BATF–JUN interaction. Cells were then transfected with the WT or MUT plasmids along with either OE-BATF or the corresponding NC plasmids, using Lipofectamine 3000 transfection reagent (Invitrogen). After 48 h of incubation, luciferase activities were assessed using the Dual-Luciferase Reporter Assay Kit (Promega).

### Animal studies

Animal experiments were conducted in accordance with the protocols approved by the Institute of Animal Care and Use Committee of the Changchun Weishi Testing Technology Service Co, Ltd. BALB/c nude mice (5–6 weeks, 18–20 g) were obtained from Hunan SJA Laboratory Animal Co, Ltd. The mice were kept under controlled environmental conditions, maintaining humidity at 55% ± 10%, a 12-h light–dark cycle, and a stable temperature of 23 ± 2 °C. A total of 24 mice were divided into four groups. HGC-27 cells (0.5 × 10^6^ cells/kg) transfected with sh-NC, sh-SLC39A5, sh-SLC39A5 + OE-NC, or sh-SLC39A5 + OE-BATF were injected subcutaneously to the right of the dorsal midline, respectively. Tumor weight and volume were measured at 0, 1, 2, 3, and 4 weeks, after which the mice were sacrificed. The GC tissues were collected for Ki-67 (ab15580, 0.1 μg/ml; Abcam) IHC staining.

To establish an orthotopic GC model, HGC-27 cells stably transduced with sh-NC, sh-SLC39A5, sh-SLC39A5 + OE-NC, or sh-SLC39A5 + OE-BATF were first injected subcutaneously (5 × 10^6^ cells per mouse) into the flanks of BALB/c nude mice. Once the subcutaneous tumors reached approximately 1 cm in maximum diameter, they were excised and cut into 1 mm^3^ tissue fragments. Each fragment was then orthotopically implanted onto the serosal surface of the greater curvature of the stomach under anesthesia, as previously described (68). Mice were monitored for body weight and signs of tumor development. At the endpoint, gastric tumors and liver tissues were collected for histological and molecular analyses, including tumor weight measurement, Western blotting for protein expression, and H&E staining to evaluate liver metastasis. Each experimental group included six mice.

### H&E staining

At the endpoint of the orthotopic tumor experiment, liver tissues were harvested from nude mice and fixed in 10% neutral-buffered formalin for 24 to 48 h. The fixed tissues were then dehydrated, embedded in paraffin, and sectioned at a thickness of 4 μm. Paraffin sections were deparaffinized in xylene, rehydrated in descending ethanol concentrations, and stained with hematoxylin (G1004; Servicebio) and eosin (G1001; Servicebio) according to the standard histological protocols. Stained sections were examined under a light microscope to assess the GC liver metastases.

### RNA extraction and qRT–PCR

Total RNA was extracted using Trizol reagent (15596026; Invitrogen). RNA (1 μg) was then converted to cDNA with the PrimeScript cDNA synthesis kit (6110A; Takara) according to the manufacturer's guidelines. qPCR was carried out using the Taqman Universal PCR Master Mix (4305719; Thermo Fisher Scientific) and primers provided by Origen Biotech as detailed in Table 3. The relative mRNA expression levels were quantified using the 2^-ΔΔCt^ method, with β-actin serving as the normalization control.

**Table 3:** Primers used for qRT–PCR

Gene	Forward sequence	Reverse sequence
SLC39A5	TCACAGGCTTCTGTTGCTGGAC	ACAGCAGAGCAAACTGACGAGG
HIP1R	AGATGCTGTGCGGAGGATTGAG	TGCAGGCTAGTGGATGTCGTCA
PIM1	GGCTCGGTCTACTCAGGCA	GGAAATCCGGTCCTTCTCCAC
JUN	TCCAAGTGCCGAAAAAGGAAG	CGAGTTCTGAGCTTTCAAGGT
β-actin	CCCTGGAGAAGAGCTACGAG	CGTACAGGTCTTTGCGGATG

### Western blot

Protein extraction was carried out with radioimmunoprecipitation assay buffer containing protease inhibitors at 4 °C for 30 min (Beyotime, Inc). Protein concentration was measured using a Bicinchoninic Acid protein assay kit (10741395; Thermo Fisher Scientific). Following this, 30 μg of protein per well was separated by SDS-PAGE and then transferred onto polyvinylidene fluoride membranes (Merck Millipore). The membranes were blocked, rinsed with PBS, and incubated with primary antibodies against SLC39A5 (PA521070, 1:1000 dilution;Thermo Fisher Scientific), PIM1 (710504, 1:100 dilution; Thermo Fisher Scientific), PIM2 (MA542509, 1:1000 dilution; Thermo Fisher Scientific), PIM3 (PA5113070, 1:1000 dilution; Thermo Fisher Scientific), BATF (135071AP, 1:1000 dilution; Thermo Fisher Scientific), HIP1R (168141AP, 1:1000 dilution; Thermo Fisher Scientific), p-PI3K (PA517387, 1:1000 dilution; Thermo Fisher Scientific), PI3K-p85 (MA174183, 1:1000 dilution; Thermo Fisher Scientific), p-AKT (700392, 1:1000 dilution; Thermo Fisher Scientific), AKT (MA514916, 1:1000 dilution; Thermo Fisher Scientific), and β-actin (MA1140, 1:5000 dilution; Thermo Fisher Scientific). After additional PBS washing, the membranes were incubated with secondary antibodies (31402, 1:2000 dilution; Invitrogen) and visualized using an Enhanced Chemiluminescence kit (WBULS0100; Merck Millipore). Results are reported as the absorbance ratio to β-actin.

### Statistical analysis

Data analysis was conducted using SPSS 20.0 software (IBM). Results are expressed as the mean ± SD from at least three independent experiments. Comparisons between two groups were made using paired/unpaired two-tailed Student's *t* test. ANOVA followed by Tukey's *post hoc* test was used for comparisons among more than two groups. Two-way ANOVA with Sidak's multiple comparisons test was performed to analyze the influence of two categorical independent variables on a continuous dependent variable. A *p* value of less than 0.05 was considered statistically significant.

## Ethics approval and consent to participate

All research protocols involving human samples were approved by the Research Ethics Review Committee of The Second Hospital of Jilin University, and informed consent was obtained from each participant.

Animal experiments were conducted in accordance with the protocols approved by the Institute of Animal Care and Use Committee of the Changchun Weishi Testing Technology Service Co, Ltd.

## Consent for publication

The informed consent is obtained from study participants.

## Data availability

All data generated or analyzed during this study are included in this published article.

## Supporting information

This article contains supporting information.

## Conflict of interest

The authors declare that they have no conflicts of interest with the contents of this article.
