# Supplementary material for: SLC39A5 promotes the malignant progression of gastric cancer by activating BATF phosphorylation
Source: J Biol Chem. 2025 Sep 22;301(11):110754. doi: 10.1016/j.jbc.2025.110754 (PMC12593631; doi:10.1016/j.jbc.2025.110754)
Supplement: Supporting Figures [file mmc1.docx]

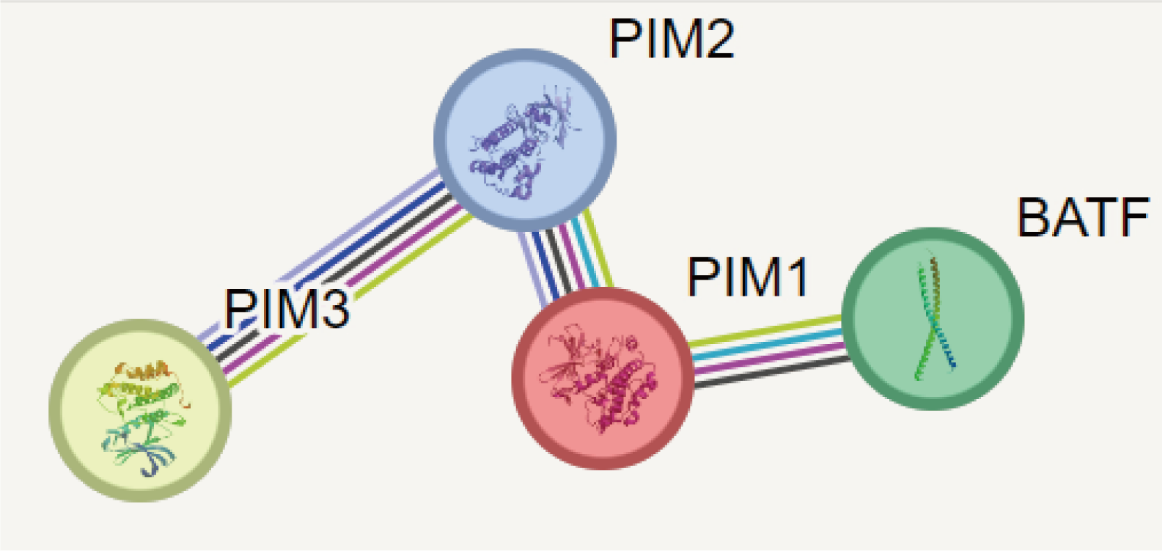


**Figure S1. Prediction of the possibility of interaction between PIMs and BATF proteins**

The possibility of protein-protein interactions between PIM1/PIM2/PIM3 and BATF was predicted by using the STRING (https://cn.string-db.org/) database. PIM, proto-oncogene serine/threonine-protein kinase Pim; BATF, basic leucine zipper transcription factor.


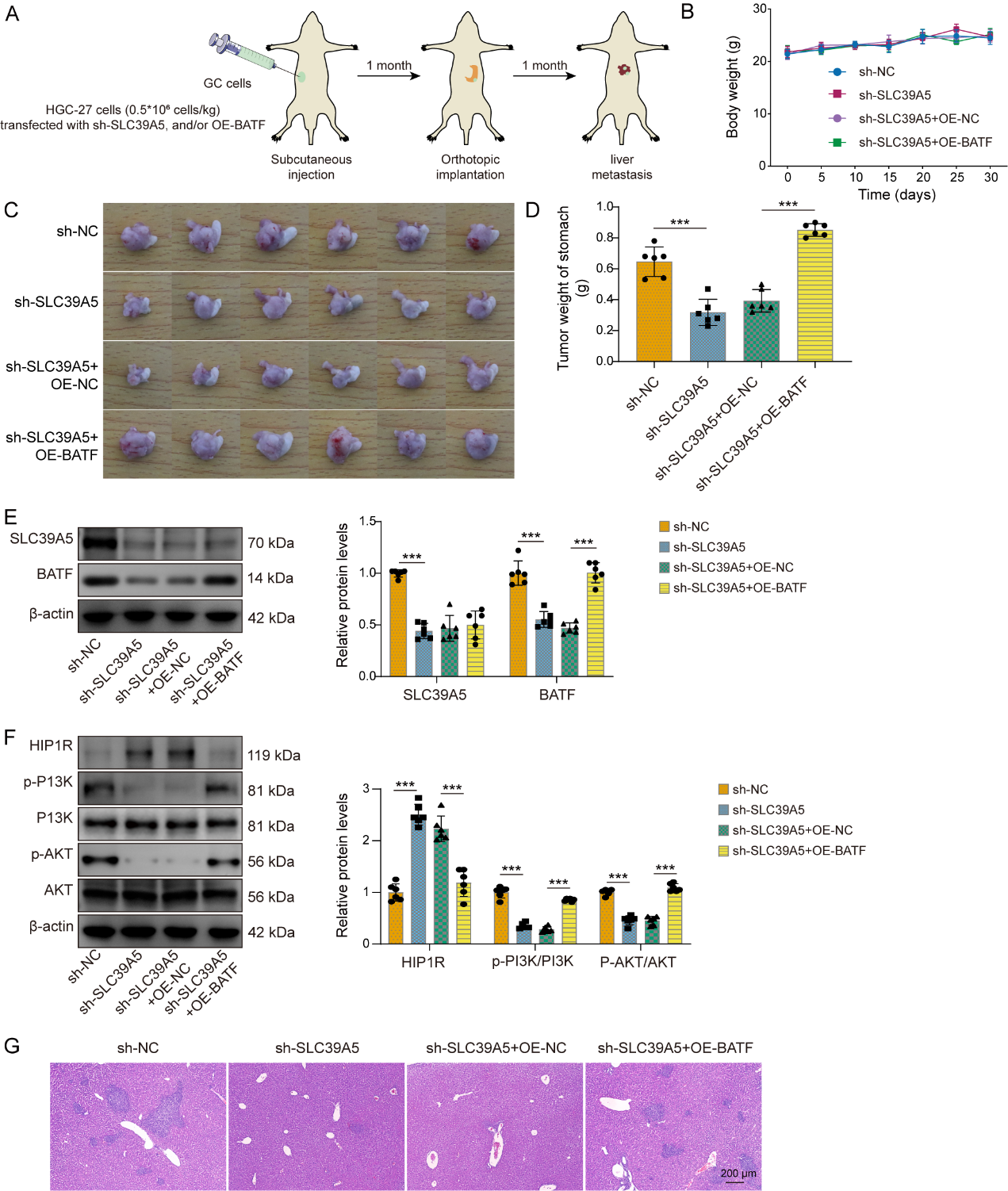
**Figure S2. SLC39A5 promoted orthotopic gastric tumor growth and liver metastasis via the BATF/HIP1R/PI3K/AKT axis.**

A total of 24 BALB/c nude mice were randomly divided into four groups (n = 6 per group). Tumor fragments derived from subcutaneous xenografts of HGC-27 cells stably expressing sh-NC, sh-SLC39A5, sh-SLC39A5 + OE-NC, or sh-SLC39A5 + OE-BATF were orthotopically implanted into the serosal surface of the greater curvature of the stomach. (A) Schematic diagram of the *in vivo* experimental design. (B) Body weight measurements of mice in each group. (C) Representative images of orthotopic gastric tumors from different experimental groups. (D) Tumor weight measurements of orthotopic gastric tumors. (E) Western blot analysis of SLC39A5 and BATF protein levels in tumor tissues. (F) Western blot analysis of HIP1R, p-PI3K, PI3K, p-AKT, and AKT in tumor tissues. (G) Representative H&E staining images of liver sections from each group, scale bar=200 μm. Data are expressed as mean±SD, n=6. For B, two-way ANOVA with Sidak's multiple comparisons test was performed to analyze data. For D-F, one-way ANOVA with Tukey's post hoc test was performed to analyze data. ***p<0.001. SLC39A5, solute carrier family 39 member 5; BATF, basic leucine zipper transcription factor; HIP1R, huntingtin-interacting protein 1-related; SD, standard deviation.
